# Interconversion Mechanisms in H_2_N–O–NH_2_: Rotamerism, Pyramidal Inversion, or Akamptisomerism?

**DOI:** 10.1021/acs.jpca.6c02943

**Published:** 2026-06-27

**Authors:** Matheus P. Freitas

**Affiliations:** † Department of Chemistry, Institute of Natural Sciences, Federal University of Lavras, 37200-900, Lavras, MG, Brazil

## Abstract

Conformational isomerism is a fundamental aspect of molecular behavior, yet bond-angle inversion (akamptisomerism) remains a rare and poorly understood mechanism, with evidence largely limited to B–O–B-bridged macrocycles. In this study, the conformational landscapes of X–O–X systems (X = B, C, N, and O) were investigated to assess the generality of this process. Conformational searches and energy profiles were computed at the GFN2-xTB and B3LYP/def2-TZVP levels. The results indicate that akamptisomerism is not a viable pathway in these systems. A linear geometry was located but corresponds to higher-order saddle points rather than a true transition state and is associated with prohibitive energy costs (>70 kcal mol^–1^). In contrast, H_2_N–O–NH_2_ undergoes interconversion via rotamerism and, more favorably, trigonal pyramidal inversion, with barriers of ∼15 and ∼8 kcal mol^–1^, respectively. These findings indicate that akamptisomerism is not a general feature of X–O–X motifs and likely requires specific geometric constraints, such as those found in macrocyclic environments.

## Graphical abstract

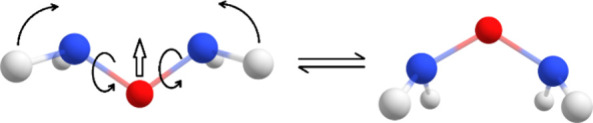

## 1 Introduction

Conformational isomerism is a fundamental topic in stereochemistry, as the dynamic spatial arrangement of atoms in a molecule governs phenomena ranging from the biological activity of drugs to the properties of materials, such as liquid crystals. While rotamerism and trigonal pyramidal inversion are well-established mechanisms of conformational interconversion, bond-angle inversion,^1^ also known as akamptisomerism, has only recently been identified.^2^ The “Hula-Twist” mechanism observed in photoreactions is likewise a relatively recent addition.^3^ In 2018, Canfield and co-workers reported that (BF)­O­(BF)-quinoxalinoporphyrin undergoes B–O–B bond-angle inversion, generating distinct stereoisomers with different B–F orientations, via a linear transition state approximately 20–25 kcal mol^–1^ above the energy minima.^2^

The proposal of akamptisomerism as a new form of stereoisomerism has attracted considerable attention because it introduces bond angle inversion as a mechanism capable of generating distinct stereochemical states. However, the extent to which this mechanism can operate in unconstrained molecular systems remains uncertain. In particular, it is not known whether the linear geometries required for bond angle inversion correspond to genuine transition states on the potential energy surface or whether they represent energetically inaccessible higher-order saddle points. Clarifying this distinction is essential for establishing the physical basis and generality of akamptisomerism.

Subsequently, two additional studies reported akamptisomerism in porphyrins and corroles,^4,5^ indicating that experimental evidence for this mechanism remains limited to these systems. In the earlier study, the authors proposed that bond-angle inversion could also occur in methoxyethane (Figure 1);^2^ however, this hypothesis remains unsubstantiated. Indeed, the role of akamptisomerism in flexible systems, such as open-chain molecules, has been largely unexplored. Therefore, the present study investigates the conformational isomerism of X–O–X systems (X = B, C, N, and O) to assess whether akamptisomerism can serve as a viable pathway for conformational interconversion and, if so, to identify the most favorable mechanism.

**Figure 1:**
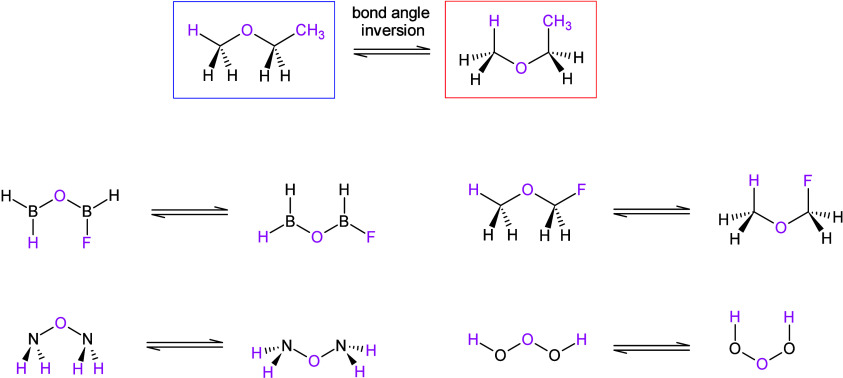
Top: Bond-angle inversion in methoxyethane as proposed by Canfield et al.^2^ Bottom: possible bond-angle inversion pathways in X–O–X systems leading to akamptisomers.

The introduction of akamptisomerism as a distinct stereochemical phenomenon raises a fundamental question regarding its scope and mechanistic basis. Although bond-angle inversion has been experimentally demonstrated in constrained B–O–B-bridged porphyrins and corroles, it remains unclear whether this process represents a general stereochemical principle or merely a consequence of the unique geometric constraints imposed by such macrocyclic frameworks. From a physical-chemical perspective, answering this question requires characterization of the corresponding potential-energy surfaces and identification of the pathways that connect stereoisomeric minima. The present study therefore investigates representative X–O–X systems (X = B, C, N, and O) to determine whether bond-angle inversion constitutes a viable interconversion mechanism and to establish the structural and energetic requirements for the occurrence of akamptisomerism.

## 2 Computational Methods

The compounds shown in Figure 1 were subjected to a conformational search using the Global Optimization Algorithm (GOAT)^6^ at the GFN2-xTB level,^7^ as implemented in ORCA.^8^ Conformers prone to undergoing akamptisomerization were subsequently optimized, and frequency calculations were performed at the B3LYP/def2-TZVP level.^9–11^ In addition, dihedral angles were scanned in 12 and 24 steps of 15°, while bond angles were scanned between stable conformers in 10° increments, passing through a linear geometry at the same level of theory, using the Gaussian 16 program.^12^ Transition-state structures were also optimized at this level.

## 3 Results and Discussion

A conformational search was performed for the target compounds; however, the equilibria depicted in Figure 1 are not observed for the boron-, carbon-, and oxygen-containing derivatives. The boron system adopts an orthogonal rather than a planar geometry, while the HO–O–OH compound exhibits three isoenergetic conformers that do not interconvert via bond-angle inversion. The fluorinated ether presents three conformers that are not interconvertible via akamptisomerism, with relative energies of 0.0, 1.8, and 4.7 kcal mol^–1^, as shown in Figure 2. These findings suggest that akamptisomerism in B–O–B-bridged porphyrins arises from the tetrahedral nature of tetracoordinated boron combined with the conformational constraints imposed by the porphyrin macrocycle. Accordingly, the present study focuses on the nitrogen-containing compound.

**Figure 2:**
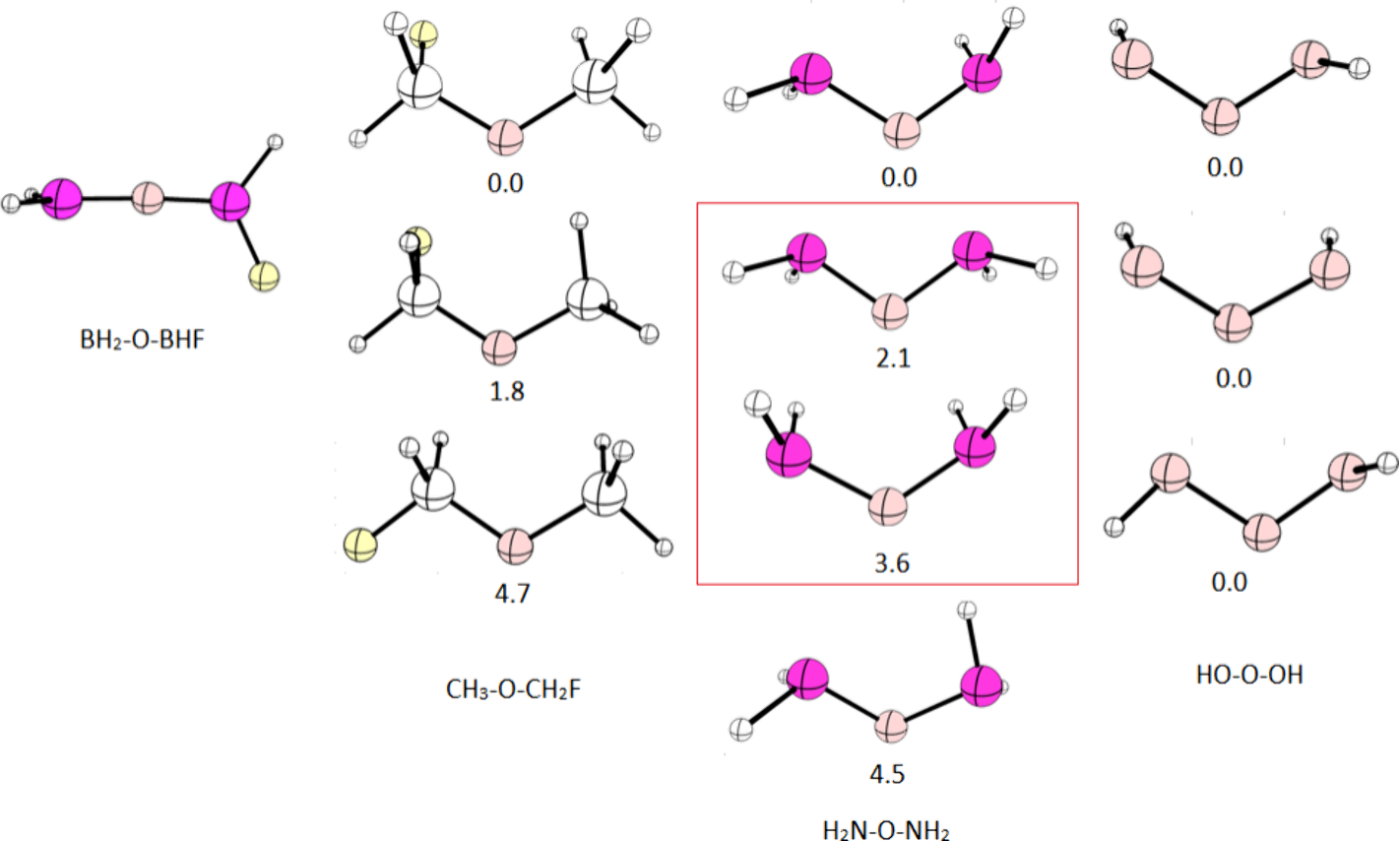
Conformers of the X–O–X systems (X = B, C, N, and O) identified from the conformational search using the Global Optimization Algorithm (GOAT) at the GFN2-xTB level (the relative electronic energies are given in kcal mol^–1^). Only the two structures highlighted for the H_2_N–O–NH_2_ compound are interconvertible via bond-angle inversion. The three conformers obtained for the boron derivative are nearly equivalent, differing only slightly in the B–O–B bond angle.

Rotation around both H–N–O–N dihedral angles in H_2_N–O–NH_2_ was investigated to evaluate the rotational barriers associated with interconversion between the two conformers (**2** and **3**) highlighted in Figure 2, which lie 2.1 and 3.6 kcal mol^–1^ above the global minimum. The potential energy surface shown in Figure 3 reveals barriers of up to ∼15 kcal mol^–1^, indicating a moderately hindered rotation. These values are higher than those typically observed for simple hydrocarbons, reflecting increased electronic interactions involving the heteroatoms, yet remain lower than the barriers characteristic of amide bonds,^1^ where partial double-bond character significantly restricts rotation. This suggests that, although conformational flexibility is reduced, rotation is still a feasible pathway for interconversion under ambient conditions.

**Figure 3:**
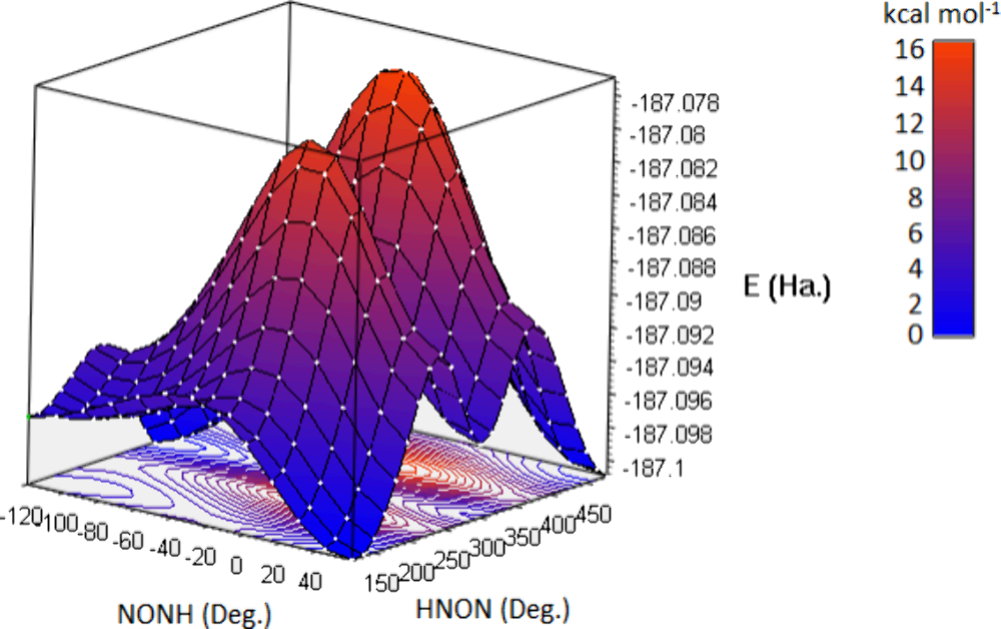
Three-dimensional potential energy surface of NH_2_–O–NH_2_, obtained at the B3LYP/def2-TZVP level.

Trigonal pyramidal inversion, also known as the umbrella effect, constitutes an alternative pathway for interconversion between forms **2** and **3** of H_2_N–O–NH_2_. To evaluate the energetic cost associated with this process, transition states corresponding to a planar NH_2_ moiety were located and are depicted in Figure 4. The calculated inversion barriers are 8.2 and 8.4 kcal mol^–1^, which are significantly lower than the maximum barrier observed for rotation around the O–N bond, indicating that inversion is the more energetically accessible pathway. This behavior can be rationalized by considering the electronic structure of nitrogen. In contrast to heavier pnictogens, such as phosphorus, the lone pair on nitrogen occupies a compact 2p orbital. The reduced spatial extension of this orbital diminishes the energetic penalty required to achieve a planar, sp^2^-like transition state, thereby facilitating rapid inversion. As a consequence, trigonal pyramidal inversion provides an efficient and viable mechanism for conformational interconversion between structures **2** and **3**, likely contributing to their dynamic equilibrium.

**Figure 4:**
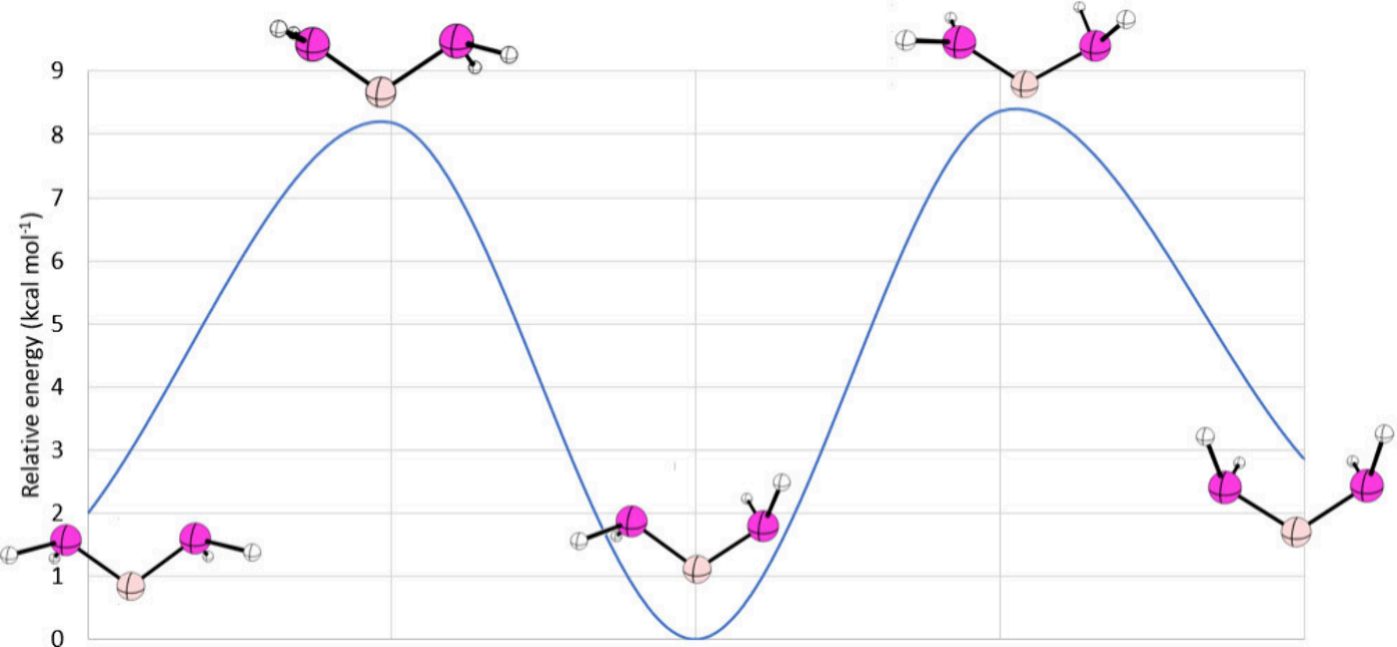
Pyramidal inversion pathway connecting structures **2** and **3**, proceeding via intermediate **1** and planar (sp^2^-like) transition states.

Finally, a third possible mechanism for interconversion between **2** and **3** involves inversion of the N–O–N bond angle, a process referred to as akamptisomerism. This pathway would require a linear transition state; however, all attempts to locate such a structure were unsuccessful, suggesting that it does not correspond to a real point on the potential energy surface. Therefore, although conceptually plausible, akamptisomerism is not operative in H_2_N–O–NH_2_. To further examine this possibility, the bond-angle inversion profile was artificially constructed by optimizing the linear structure connecting species **2** and **3**. This structure corresponds to a saddle point with three imaginary frequencies, one of which (−952.05 cm^–1^) is associated with bond-angle inversion. The resulting energy profile (Figure 5) clearly indicates that akamptisomerism is energetically prohibitive, as linearization requires more than 70 kcal mol^–1^—an energy cost comparable to that of a π bond. This barrier is significantly higher than that in water, for which the bond-angle inversion barrier, obtained from the difference in standard Gibbs free energies between the bent minimum and the linear transition state (imaginary frequency at −1607.02 cm^–1^), is 29.2 kcal mol^–1^. According to Walsh,^13,14^ widening of the bond angle significantly perturbs the energies of the oxygen s and p orbitals involved in bonding. As the structure approaches linearity, the p orbital aligned with the N–O bonds is stabilized due to improved frontal overlap. In contrast, the remaining orbitals experience a substantial increase in energy when progressing from the equilibrium bent geometry to the linear configuration. This opposing trend arises because, in the bent structure, the HOMO retains some bonding character, whereas upon linearization, rehybridization increases its p-character and diminishes the effectiveness of orbital overlap. In other words, the loss of efficient orbital overlap in the linear geometry destabilizes the system, making this inversion pathway energetically inaccessible.

**Figure 5:**
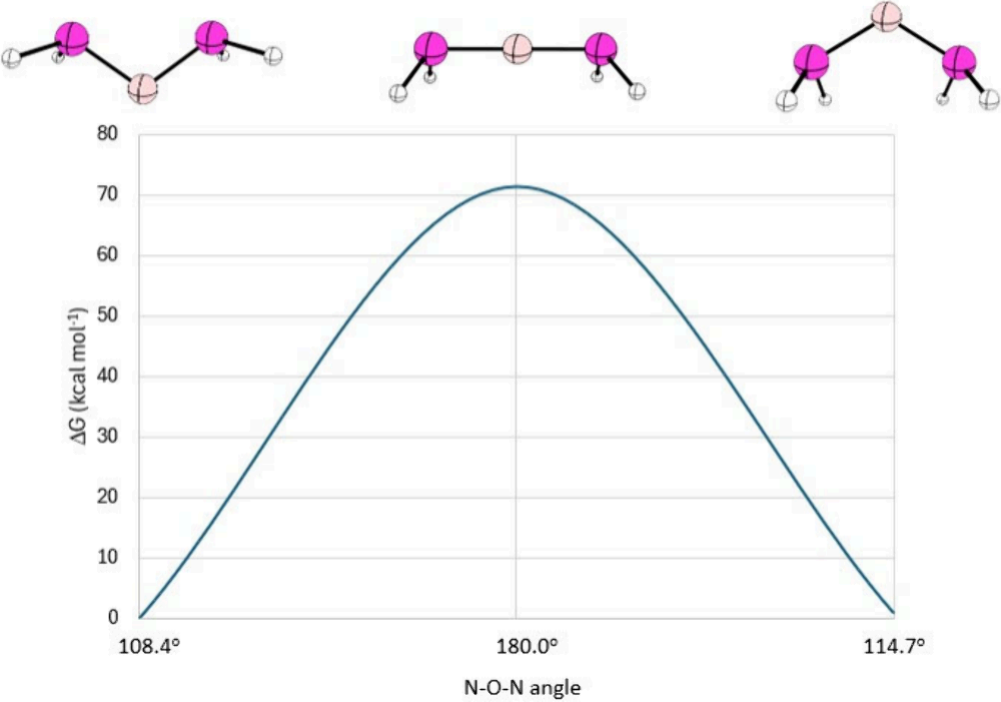
N–O–N bond angle inversion pathway connecting structures **2** and **3**, reaching a maximum at a hypothetical linear configuration.

## 4 Conclusions

In summary, the interconversion between forms **2** and **3** of H_2_N–O–NH_2_ follows the energetic order pyramidal inversion < rotamerism ≪ akamptisomerism. In practice, akamptisomerism does not occur, being thus far limited to B–O–B-bridged porphyrins and corroles. Since the boron-, carbon-, and oxygen-based derivatives examined here do not exhibit conformers capable of interconverting via bond-angle inversion, bond rotation remains the only feasible pathway in these systems. Nevertheless, it is important to consider that macrocyclic environments can impose significant constraints on bond rotation. Given that tetrahedral boron undergoes akamptisomerization in the aforementioned systems, the incorporation of alternative bridges—such as ethers, trioxidanes, and diazoxanes—into porphyrin frameworks may provide promising structural motifs for enabling this rare form of conformational isomerism.

## Supplementary Material



## Notes

Coordinates and absolute energies of the optimized structures (PDF)
