# Supplementary material for: Interconversion Mechanisms in H2N–O–NH2: Rotamerism, Pyramidal Inversion, or Akamptisomerism?
Source: J Phys Chem A. 2026 Jun 27;130(27):5222–5. doi: 10.1021/acs.jpca.6c02943 (PMC13359359; doi:10.1021/acs.jpca.6c02943)
Supplement: Supplementary file 1 [file jp6c02943_si_001.pdf]

SUPPORTING INFORMATION

Interconversion Mechanisms in $H_2N–O–NH_2$: Rotamerism, Pyramidal Inversion, or Akamptisomerism?

Matheus P. Freitas[a]*

[a] Department of Chemistry, Institute of Natural Sciences, Federal University of Lavras, 37200-900, Lavras, MG, Brazil

* E-mail: matheus@ufla.br

Pages 2-8. Geometries and energies obtained for BF_2-O-BHF, CH_3-O-CH_2F, NH_2-O-NH_2, and HO-O-OH.

Geometries and energies obtained from GOAT at the GFN2-xTB level

BH_2-O-BHF **1**

8	0.490771753	1.315880198	0.051407696
5	1.571338417	0.614245741	-0.145317796
1	2.570627106	0.935851886	0.388816916
1	1.521040065	-0.337076404	-0.837939565
5	-0.567800996	2.052070774	0.192105763
1	-1.368925625	1.897486208	1.037219480
9	-0.767050720	3.051541596	-0.675292495

E = -12.93967719040 hartrees

BH_2-O-BHF **2**

8	0.505099940	1.337082442	0.047611595
5	1.571329734	0.612948221	-0.144913507
1	1.505324146	-0.335945891	-0.839536409
1	2.574563907	0.915031335	0.392911771
5	-0.565070038	2.055585655	0.190154508
1	-1.365021942	1.885260205	1.033437240
9	-0.776225748	3.060038031	-0.668665198

E = -12.93965704440 hartrees

BH_2-O-BHF **3**

8	0.627746935	1.547814769	-0.262076572
5	1.543065197	0.616732757	-0.115401122
1	2.499839982	0.851250314	0.528860362
1	1.421887161	-0.404069219	-0.689103588
5	-0.504705251	2.087700984	0.098175461
1	-1.136552913	1.703591711	1.015414515
9	-1.001281111	3.126978684	-0.564869056

E = -12.93485910990 hartrees

CH_3-O-CH_2F **1**

8	-0.034880336	0.746067555	-0.110884034
9	0.072243347	2.735997246	-1.193272236
6	-0.458590740	2.040418884	-0.125777635
1	-0.157579908	2.608773908	0.772002739
1	-1.545752651	2.040182855	-0.256213235
6	1.345037350	0.587627287	0.177091464
1	1.587143075	1.017096128	1.155415984
1	1.531255236	-0.483126108	0.186611347
1	1.962124628	1.062962244	-0.589974392

E = -15.62050308400 hartrees

CH_3-O-CH_2F **2**

8	0.067313890	0.823034936	-0.412751048
9	0.135599905	3.066609309	-0.732460264
6	-0.492231433	2.018503058	-0.086792772
1	-0.431894012	2.261339917	0.988106126
1	-1.530264786	2.008766353	-0.436161459
6	1.383887661	0.595643679	0.083199759
1	1.758233695	1.458009079	0.635397744
1	1.364966614	-0.278188528	0.738314087
1	2.045388467	0.402282197	-0.761852174

E = -15.61769864900 hartrees

CH_3-O-CH_2F **3**

8	0.350526118	0.976077866	-0.676780939
9	-1.153731076	2.478694377	-1.287791513
6	-0.290689994	2.144708622	-0.302830109
1	0.418276209	2.985846495	-0.185062322
1	-0.880981106	2.014389099	0.623772343
6	1.280603341	0.534245491	0.281063513

1	0.792572459	0.322377547	1.239863439
1	1.714626112	-0.378943252	-0.117937381
1	2.069797938	1.278603755	0.440702970

E = -15.61294013670 hartrees

NH_2-O-NH_2 **1**

8	-1.144254917	1.599968825	0.492662526
7	-0.982948064	2.757508266	1.238928518
1	-1.674640657	3.449501471	0.954059240
1	-1.105694763	2.548478050	2.228720266
7	-2.451585915	1.016511656	0.663545609
1	-2.867020508	0.965108302	-0.263570640
1	-2.301855177	0.069923430	1.004654481

E = -11.82491424930 hartrees

NH_2-O-NH_2 **2**

8	-1.321101736	1.648250931	0.603181853
7	-1.474954360	2.766704230	1.466510350
1	-0.749459752	2.695993105	2.174590122
1	-1.325796306	3.598834510	0.902536949
7	-2.522408385	0.888767925	0.606302504
1	-2.854091403	0.855585166	-0.353700422
1	-2.280188058	-0.047135867	0.919578644

E = -11.82155221600 hartrees

NH_2-O-NH_2 **3**

8	-0.998443890	1.565152363	0.401330566
7	-1.066115832	2.692452404	1.229847202
1	-1.803678414	3.322727162	0.915676408
1	-1.229586437	2.415217197	2.197500642
7	-2.138701719	0.754304197	0.338308532

1	-2.358482564	0.374871661	1.259003081
1	-2.932991144	1.282275016	-0.022666431

E = -11.81921702270 hartrees

NH_2-O-NH_2 **4**

8	-1.419177777	1.808392904	0.291978843
7	-0.979859449	2.642503701	1.390274805
1	-0.932493306	3.575672701	0.980414852
1	-1.746078052	2.638597247	2.066033641
7	-2.390094833	0.893362096	0.748598448
1	-3.111551318	0.871474299	0.032431359
1	-1.948745265	-0.023002949	0.809268053

E = -11.81777758030 hartrees

HO-O-OH **1**

8	-1.804414619	2.463460522	-0.189536153
8	-1.462402827	2.849957405	1.101775811
1	-0.491546806	2.858583237	1.133370150
8	-1.879342294	1.077351804	-0.272377585
1	-2.789293454	0.840647032	-0.028232222

E = -13.04911338830 hartrees

HO-O-OH **2**

8	-1.923598541	2.533339523	-0.157017744
8	-0.789934816	2.787579265	0.606314095
1	-1.076664766	2.799989411	1.535195245
8	-2.021576847	1.178418488	-0.452304676
1	-2.615225030	0.790673311	0.212813080

E = -13.04416070620 hartrees

HO-O-OH **3**

8	-1.952557330	2.470792865	-0.046199475
8	-1.345468863	3.038134879	1.040233099
1	-0.419980397	2.734423821	1.039479002
8	-2.346192455	1.139810002	0.287402558
1	-2.362800955	0.706838433	-0.575915184

E = -13.04290208150 hartrees

Geometries optimized at the B3LYP/def2-TZVP level and Gibbs free energies

H_2N-O-NH_2 **1**

8	-1.134265000	1.590055000	0.481849000
7	-0.958168000	2.767042000	1.234626000
1	-1.696980000	3.410826000	0.953513000
1	-1.137505000	2.524440000	2.208444000
7	-2.460749000	1.045685000	0.688493000
1	-2.850143000	0.977952000	-0.250208000
1	-2.291190000	0.092000000	1.003283000

G^o = -186.97376286 hartrees

H_2N-O-NH_2 **2**

8	-1.623319000	2.166951000	0.015004000
7	-1.085882000	2.638474000	1.254859000
1	-0.079289000	2.638451000	1.105863000
1	-1.382949000	3.610582000	1.301466000
7	-2.439479000	1.022022000	0.282038000
1	-3.345891000	1.265176000	-0.111303000
1	-2.043193000	0.291345000	-0.304926000

G^o = -186.97059017 hartrees

H_2N-O-NH_2 **3**

8	-1.619254000	2.170873000	0.002847000
7	-1.053953000	2.698034000	1.193083000
1	-1.822902000	2.937999000	1.819679000
1	-0.510196000	1.954168000	1.631828000
7	-2.446216000	1.033131000	0.191953000
1	-1.881844000	0.313979000	0.645628000
1	-3.193636000	1.298815000	0.833982000

G^o = -186.96920134 hartrees

H_2N-O-NH_2 **TS1**

8	-1.729531000	1.227155000	-0.262058000
7	-2.074563000	2.658955000	-0.230850000
1	-2.545779000	2.806875000	-1.122996000
1	-2.802908000	2.691065000	0.480986000
7	-0.380127000	1.059049000	-0.259735000
1	0.067878000	0.949573000	0.629149000
1	0.068030000	0.936327000	-1.146496000

G^o = -186.96071825 hartrees (img. freq. = 852.67 cm^{-1})

H_2N-O-NH_2 **TS2**

8	-1.626060000	2.184241000	-0.017948000
7	-0.967830000	2.857725000	1.075219000
1	-1.707668000	3.106939000	1.731792000
1	-0.400316000	2.146409000	1.536288000
7	-2.372662000	1.098455000	0.375023000
1	-1.914825000	0.207190000	0.407431000
1	-3.332638000	1.258041000	0.616195000

G^o = -186.96042324 hartrees (img. freq. = 856.04 cm^{-1})
